# Quantitative Evaluation of Therapeutic Response by FDG-PET–CT in Metastatic Breast Cancer

**DOI:** 10.3389/fmed.2016.00019

**Published:** 2016-05-09

**Authors:** Dorothée Goulon, Hatem Necib, Brice Henaff, Caroline Rousseau, Thomas Carlier, Françoise Kraeber-Bodere

**Affiliations:** ^1^Service de médecine nucléaire, ICO Nantes, Saint Herblain Cedex, France; ^2^Service de radiologie, CHU Nantes, Nantes, France; ^3^Service de médecine nucléaire, CHU Nantes, Nantes, France; ^4^INSERM UM R892, Nantes, France

**Keywords:** FDG, PET, breast cancer, PERCIST, therapeutic evaluation, parametric analysis, SULTAN

## Abstract

**Purpose:**

To assess the therapeutic response for metastatic breast cancer with ^18^F-FDG position emission tomography (PET), this retrospective study aims to compare the performance of six different metabolic metrics with PERCIST, PERCIST with optimal thresholds, and an image-based parametric approach.

**Methods:**

Thirty-six metastatic breast cancer patients underwent 128 PET scans and 123 lesions were identified. In a per-lesion and per-patient analysis, the performance of six metrics: maximum standardized uptake value (SUVmax), SUVpeak, standardized added metabolic activity (SAM), SUVmean, metabolic volume (MV), total lesion glycolysis (TLG), and a parametric approach (SULTAN) were determined and compared to the gold standard (defined by clinical assessment and biological and conventional imaging according RECIST 1.1). The evaluation was performed using PERCIST thresholds (for per-patient analysis only) and optimal thresholds (determined by the Youden criterion from the receiver operating characteristic curves).

**Results:**

In the per-lesion analysis, 210 pairs of lesion evolutions were studied. Using the optimal thresholds, SUVmax, SUVpeak, SUVmean, SAM, and TLG were significantly correlated with the gold standard. SUVmax, SUVpeak, and SUVmean reached the best sensitivity (91, 88, and 83%, respectively), specificity (93, 95, and 97%, respectively), and negative predictive value (NPV, 90, 88, and 83%, respectively). For the per-­patient analysis, 79 pairs of PET were studied. The optimal thresholds compared to the PERCIST threshold did not improve performance for SUVmax, SUVpeak, and SUVmean. Only SUVmax, SUVpeak, SUVmean, and TLG were correlated with the gold standard. SULTAN also performed equally: 83% sensitivity, 88% specificity, and NPV 86%.

**Conclusion:**

This study showed that SUVmax and SUVpeak were the best parameters for PET evaluation of metastatic breast cancer lesions. Parametric imaging is helpful in evaluating serial studies.

## Introduction

Metastatic breast cancer is initially diagnosed in 6–10% of cases and during follow-up in 30% of cases (1). The treatment strategy in this situation is mainly based on chemotherapy, hormonal therapy, targeted therapies, and possibly external radiotherapy. The accurate and early assessment of therapeutic efficacy represents a major challenge but is crucial for limiting toxicity and reducing expensive treatments.

Current therapeutic responses for solid tumors are conventionally assessed using the international standard RECIST 1.1 (2). However, RECIST has a number of intrinsic limitations such as moderate reproducibility of tumor measurement (3), late occurrence of morphological response compared to early metabolic changes, not applicable with non-measurable morphological lesions (bone lesions, lymphangitis, and effusions), and in targeted cytostatic therapies. Functional imaging by position emission tomography (PET) with 18-fluorodeoxyglucose (^18^FDG) represents a potential alternative (4, 5). Specific evaluation criteria for metabolic responses have been previously defined. These include measures of quantitative metrics and visual analysis tools to classify tumor progression and response, as defined by the European Organization for Research and Treatment of Cancer (EORTC) (6) or PERCIST (3).

The ^18^FDG-PET showed interest in breast cancer management (7), for initial staging of locally advanced cancers (stages II–III) and/or inflammatory lesions (8), detection of recurrence with better performance than conventional imaging (7, 9, 10), evaluation of therapeutic response to neo-adjuvant therapy in inoperable locally advanced cancers or before conservative surgery or inflammatory lesions (7, 11), and therapy evaluation in metastatic disease (5, 12–18). However, although ^18^FDG-PET proved interest in several clinical studies, it is not used in clinical practice for therapy assessment because of the lack of standardization of imaging interpretation (12). Some studies suggested a benefit of using semi-quantitative analysis (mainly the change in SUVmax or SUVmean between two PET scans) rather than visual analysis only. However, the best metric and optimal threshold was not clearly defined. Moreover, it is worth noting that none of these studies were based on the PERCIST approach proposed by Wahl et al. (3).

Semi-quantitative methods (3–6) have been proposed for therapeutic evaluation using PET to improve reproducibility based on the percentage variation of a metric (SUVmax for EORTC and SULpeak for PERCIST). Yet, they have not been validated in the context of specific tumors, especially breast cancer (12). Moreover, some requirements of PERCIST (mainly need for a tumor size >2 cm and no difference between liver signal between the two PET scans) may be difficult to achieve in clinical practice.

New evaluation methods based on parametric analysis are also being developed, while the best metrics and optimal thresholds were not clearly defined (19). The SULTAN (longitudinal monitoring in tomography using factor analysis) method, for example, proposes a novel semi-automatic method to assist in tumor response assessment by studying the metabolic change at the voxel level (20, 21). SULTAN provides a parametric map of the tumor metabolic change using two or more PET scans and allows the heterogeneity of response within the tumor to be determined.

The first objective of this retrospective study was to compare the performance of different metabolic metrics on a per-lesion and per-patient basis in the assessment of therapeutic response in metastatic breast cancer.

The second objective was to assess the benefit of parametric imaging (SULTAN) in this population.

## Materials and Methods

### Patients and Imaging Protocols

For this single center study conducted from September 2009 to July 2014, 36 patients (median age 63.5 years, range: 39–85 years) with breast cancer of any histological grade and metastatic involvement (i.e., initially metastatic or metastatic following diagnostic evaluation), underwent at least two ^18^FDG-PET scans using the same PET system in the course of their therapy. Tumor phenotypes were classified as 26 invasive ductal carcinomas, 6 invasive lobular carcinomas, 3 intraductal carcinomas, and 1 colloid carcinoma. Twenty-eight tumors were estrogen receptor (ER) positive, 21 progesterone receptor (PR) positive, 4 HER2 over-expression (HER2), and 6 were triple-negative. Treatments consisted of adjuvant chemotherapy, hormonal therapies, targeted therapies, Herceptin, and/or radiotherapy. A total of 128 PET scans were acquired (median of 3 PET/patient, range: 2–9) with a median time interval of 3.7 months between two PET (range: 1.1–19.6). A total of 123 lesions were analyzed: 44 lymph nodes, 43 bone lesions, 17 liver lesions, 10 breast lesions, 5 lung lesions, and 4 peritoneal carcinomatosis. A total of 79 pairs of PET scans were analyzed in 36 patients.

Position emission tomography scans were conducted in patients fasted for at least 6 h, with normal blood glucose <10 mmol/L, 1 h after injection of 3 or 7 MBq/kg of ^18^FDG (depending on the PET system used), using either a Siemens Biograph mCT 40 camera (Siemens Healthcare Molecular Imaging USA, Inc.) or a General Electric Discovery LS (GE Medical Systems, Waukesha, WI, USA). The low-dose computed tomography acquisition was performed first without injection of iodinated contrast agent, followed by PET acquisition using 3 min per bed position (Siemens Biograph mCT) or 5 min for the GE Discovery LS. The following acquisition constraints according to the PERCIST framework were respected: similar activity between each PET scan (±20%), standardization against normal liver, and a similar delay between injection and acquisition (50–70 min after injection).

### Image Analysis Using Semi-Quantitative Metrics

Six PET-based metrics were derived, for a maximum of five tumor targets (maximum of two targets per organ) as recommended by PERCIST (3): SUVmax, SUVpeak, SUVmean, metabolic volume (MV), total lesion glycolysis (TLG = SUVmean × MV), and standardized added metabolic activity (SAM) (22). SAM was proposed to overcome the partial volume effect. The segmentation approach proposed by Schaefer was used for computing SUVmean, MV, and TLG (23).

The gold standard was defined by clinical assessment, and biological and conventional imaging by CT and MRI, performed 3 weeks after the PET evaluation. RECIST 1.1 (2) was used in these assessments. Each evolution was classified as either a responder or non-responder according to the gold standard.

A “responder” as assessed by PET was defined as a metric decrease greater than the threshold, while a “non-responder” was defined as a decrease of less than the threshold or an increase in the metrics. The four different types of response were true positive (TP), responder according to PET and the gold standard; true negative (TN), non-responder according to PET and the gold standard; false negative (FN), non-responder according to PET but responder according to the gold standard; and false positive (FP), responder according to PET but non-responder according to the gold standard.

### Image Analysis Using Parametric Imaging (SULTAN)

SULTAN is a parametric approach that compares two or more PET scans acquired before and during therapy (20, 21). In the context of this study, pairs of PET volumes acquired for the same patient were considered.

This new approach involves a rigid registration between the two PET scans, followed by a factor analysis as briefly described in the following sections.

#### Registration of PET Volumes

To compare two PET images at a voxel level, these scans first need to be registered so that a given voxel corresponds to the same volume element in each of the two scans. The method used was described in Ref. (24). Briefly, the CT volumes were used to determine the appropriate transformation for aligning the PET images, as they include far more anatomical details for guiding registration than the PET images. The two CT volumes of interest (VOIs) were registered using a rigid transformation (three translation and three rotation parameters) derived from block-matching registration (19) as implemented in the Planet Onco software (Dosisoft). Local rigid transformation was assumed as only the region including mass was actually registered. The transformation mapping the second CT volume onto the first CT volume was then applied to the second PET scan so as to align it with the first PET scan, assuming the PET and CT of a given scan were perfectly registered.

#### Calculation of Parametric Image of Significant Tumor Changes

The two registered PET scans, denoted as PET1 and PET2, were analyzed using a factor analysis of dynamic sequences (FADS) approach (25) as implemented in the software Pixies [Apteryx, 2004]. The algorithm assumes that the two-component vector *S*(*v*, *t*) measured in each voxel (one value for the first scan and one value for the second scan) is a weighted sum of *K* basis functions. In this algorithm, the number *K* is constrained by the number of PET scans, hence is equal to 2. Let *S*(*v*, *t*) be the signal recorded at the voxel *v* for the time *t* (*t* = 1, 2). Then,

(1) $$S(v,t)=I_{b}(v).C_{b}(t)+I_{e}(v).C_{e}(t)+e(v,t)$$

where *C*_b_(*t*) and *C*_e_(*t*) are two basis kinetics, *I*_b_ is the spatial distribution of the voxel component following the *C*_b_ time course, *I*_e_ is the spatial distribution of the voxel component following the *C*_e_ time course, and *e*(*v*, *t*) is an additive error term. Factor analysis estimates the two functions *C*_b_(*t*) and *C*_e_(*t*), called factors, and their associated images *I*_b_(*v*) and *I*_e_(*v*), called factor images.

Equation 1 is solved using a principal component analysis followed by an oblique rotation under a constant function constraint representing the constant voxels of the background (*C*_b_) and without any other constraint on *I*_b_(*v*) and *I*_e_(*v*). The algorithm iteratively estimates the two factors, *C*_b_ and *C*_e_, and the associated factor images, *I*_b_ and *I*_e_ (25). Therefore, the voxels that evolved between the two scans followed the *C*_e_ factor.

A new image (SULTAN image) is then created whereby each pixel *v* is equal to *I*_e_(*v*) if |*I*_e_(*v*)| > 1 or 0 otherwise. Hence, each voxel reflects its evolution over time following the *C*_e_ factor (*I*_e_ > 0) or the opposite direction of *C*_e_ (*I*_e_ < 0).

Finally, each lesion was classified as responder (main factor decreasing with *I*_e_ > 0 or main factor increasing with *I*_e_ < 0) or non-responder (main factor increasing with *I*_e_ > 0 or main factor decreasing with *I*_e_ < 0). Patient was considered as responder if all lesions were responders and non-responder otherwise. The results were then classified as VP, VN, FP, and FN by comparison with the gold standard.

### Statistical Analysis

The study was performed using a per-lesion and a per-patient analysis. For each analysis, the metrics were compared using the area under the curve (AUC) determined with receiver operating characteristic (ROC) analysis.

The optimal thresholds were derived using the Youden criterion [max (sensitivity + specificity − 1)] through the ROC analysis for the per-lesion and per-patient studies.

The per-lesion analysis was performed using the percentage change using optimal threshold of each metabolic metric. Each lesion was then compared with the gold standard.

The per-patient analysis was performed using the PERCIST criteria (the percentage change of each metabolic metric for the most intense lesion in each PET between two scans). The percentage change was interpreted as responder or non-responder using previously optimized thresholds but also using PERCIST threshold (30% for each metric, except 45% for TLG). Each pair of PET scans was then compared with the gold standard.

The sensitivity, specificity, positive predictive value (PPV), negative predictive value (NPV), and accuracy were then calculated for each index.

Pearson’s chi-squared analysis with a type I error of 0.05 and 1 degree of freedom was performed to determine significant associations between the different quantitative metrics and the gold standard.

Statistical significance was set to *p* < 0.05. Statistical analysis was performed using MedCalc Statistical Software version 14.12.0 (MedCalc Software, Ostend, Belgium; http://www.medcalc.org; 2014).

We obtained informed consent from all patients allowing the use of their clinical data for research purposes under a protocol approved in our institution.

## Results

### Per-Lesion Analysis Using Quantitative Metrics

A total of 123 lesions and 210 pairs of lesion evolutions, followed on two to nine scans, were analyzed with 111 considered as responders and 99 as non-responders according to the gold standard.

**Figure 1:**
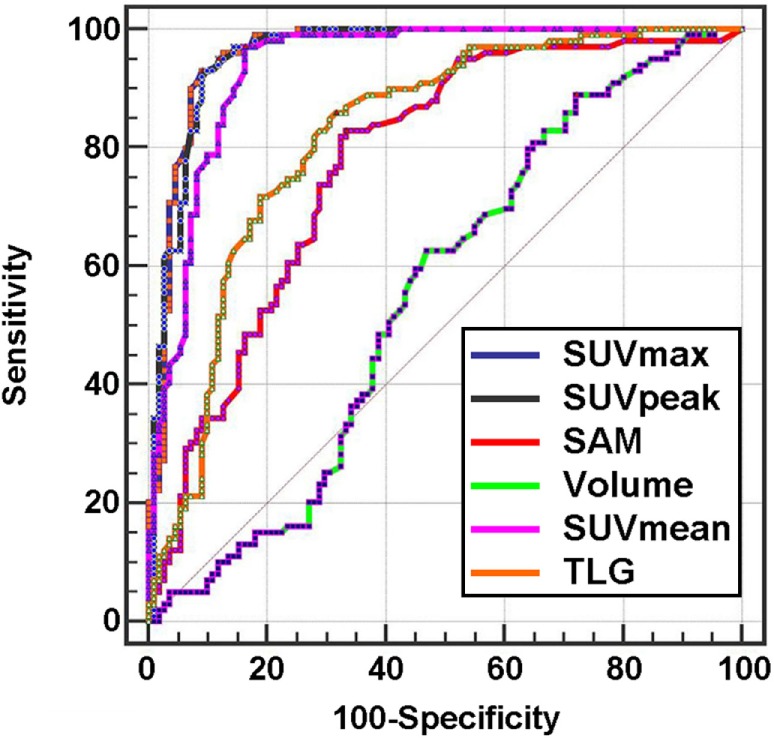
**ROC curves of metabolic indices for per-lesion analysis**.

**Figure 2:**
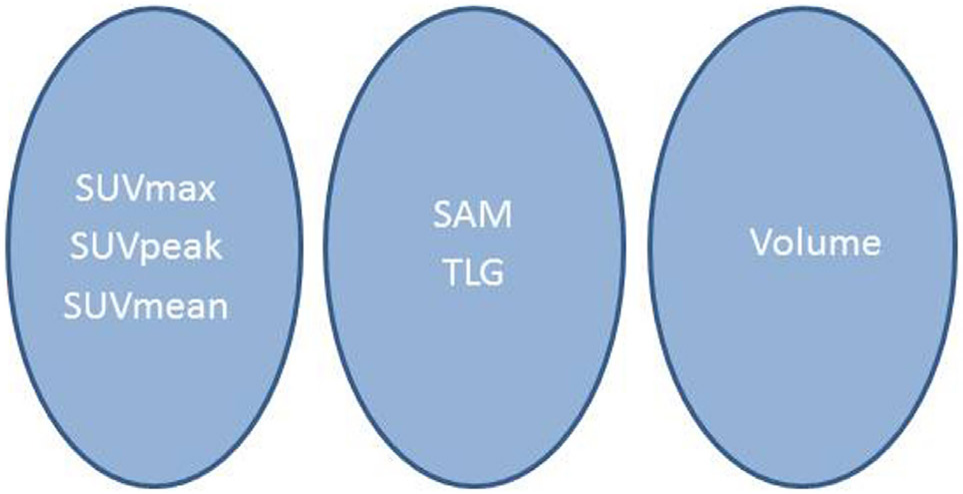
**Synthetic scheme of the results of the intercomparison per-lesion study**. Indices lying in the same circle were not significantly different.

Figure 1 shows the results of the ROC study for the six metrics. The AUC values (Table 1) ranged from 0.55 for MV to 0.96 for SUVmax. The AUC intercomparison study distinguished three significantly distinct groups: SUVmax/SUVpeak/SUVmean, SAM/TLG, and MV (Figure 2).

**Table 1:** **Metabolic metrics AUC for per-lesion analysis**.

Metrics	SUVmax	SUVpeak	SUVmean	SAM	MV	TLG
AUC	0.960	0.958	0.937	0.775	0.554	0.822

The optimal thresholds defined by the Youden criterion, were 21% for SUVmax, 23% for SUVpeak, 29% for SUVmean, 48% for SAM, 33% for MV, and 20% for TLG.

Sensitivity, specificity, PPV, NPV, accuracy values, and Youden correlation coefficients were calculated for their optimal threshold (Table 2).

**Table 2:** **Comparison of metabolic metrics for per-lesion analysis with optimal thresholds**.

Metrics	SUVmax	SUVpeak	SUVmean	SAM	MV	TLG	SULTAN
Threshold (%)	−21	−23	−29	−48	−33	−20	
Sensitivity (%)	91	88	83	66	27	68	86
Specificity (%)	93	95	97	83	89	86	75
PPV (%)	94	95	97	81	73	84	79
NPV (%)	90	88	83	68	52	70	83
Accuracy (%)	92	91	90	74	56	76	81
Youden correlation coefficient	0.84	0.83	0.80	0.49	0.16	0.53	0.61
Significance (χ^2^*p* < 0.05)	S	S	S	S	NS	S	S

*Significance: correlation between the metric and the gold standard according to Youden correlation coefficient*.

Five metrics (SUVmax, SUVpeak, SUVmean, SAM, and TLG) significantly correlated with the gold standard (*p* < 0.05), but the analysis of correlation coefficients (Youden index) showed that SUVmax, SUVpeak, and SUVmean led to the best performance in terms of sensitivity (91, 88, and 83%, respectively), specificity (93, 95, and 97%, respectively), and NPV (90, 88, and 83%, respectively).

### Per-Patient Analysis Using Quantitative Metrics

A total of 79 pairs of PET scans were analyzed using the PERCIST criteria (the most intense lesion in each PET between two scans) with 36 responders and 43 non-responders.

**Figure 3:**
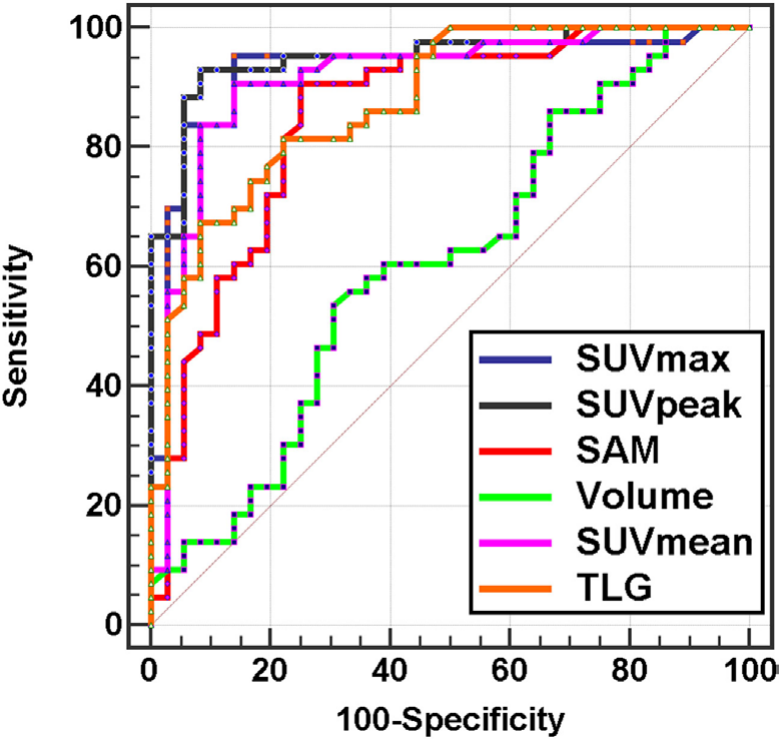
**ROC curves of metabolic indices for per-patient analysis**.

**Figure 4:**
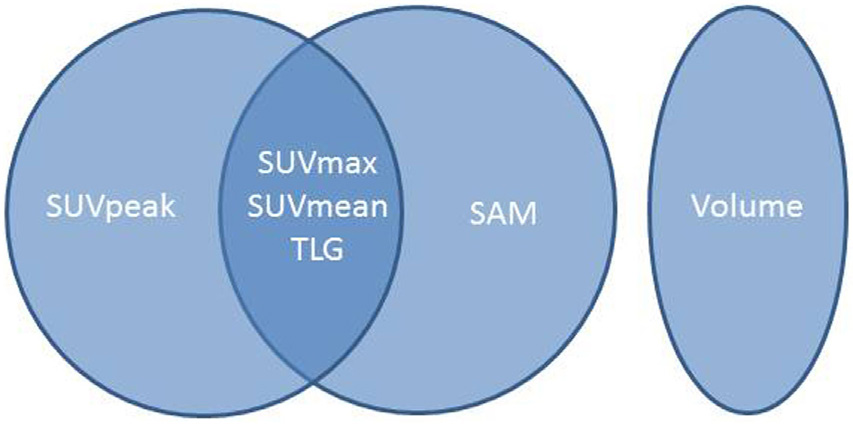
**Synthetic scheme of the results of the intercomparison per-patient study**. Indices lying in the same circle were not significantly different.

The AUC (Figure 3; Table 3) ranged from 0.61 for MV to 0.95 for SUVpeak. The AUC of SUVpeak, SUVmax, SUVmean, TLG, and SAM were significantly different from MV (*p* < 0.05) but not between each other (Figure 4).

**Table 3:** **Metabolic metrics AUC for per-patient analysis**.

Metrics	SUVmax	SUVpeak	SUVmean	SAM	MV	TLG
AUC	0.928	0.952	0.914	0.851	0.606	0.876

The percentage change of each metabolic metric was also interpreted as responder or non-responder according to the choice of the threshold (PERCIST or optimal) and then compared with the gold standard.

With PERCIST thresholds (30% for each metric, except 45% for TLG), only SUVmax, SUVpeak, and SUVmean were significantly correlated with the gold standard (*p* < 0.05) (Table 4).

**Table 4:** **Comparison of metabolic metrics for per-patient analysis according to PERCIST threshold**.

Metrics	SUVmax	SUVpeak	SUVmean	SAM	MV	TLG
Threshold (%)	−30	−30	−30	−30	−30	−45
Sensitivity (%)	75	67	67	39	8	36
Specificity (%)	95	98	95	95	100	100
PPV (%)	93	96	92	88	100	100
NPV (%)	82	78	77	65	57	65
Accuracy (%)	86	84	82	70	58	71
Youden correlation coefficient	0.70	0.64	0.62	0.34	0.08	0.36
Significance (χ^2^* p* < 0.05)	S	S	S	NS	NS	NS

The best thresholds were 36% for SUVmax, 26% for SUVpeak, 29% for SUVmean, 54% for SAM, 58% for MV, and 27% for TLG.

After applying these optimized thresholds, the four metrics (SUVpeak, SUVmax, SUVmean, and TLG) were correlated with the gold standard (Table 5). Threshold optimization did not change the specificity of SUVmax (98 vs. 95%). The sensitivity using SUVpeak was slightly improved (72 vs. 67%) with a similar NPV (81 vs. 78%). The sensitivity, NPV, and accuracy of TLG were improved (53 vs. 36%, 72 vs. 65%, and 78 vs. 71%, respectively).

**Table 5:** **Comparison of metabolic metrics and SULTAN for per-patient analysis according to optimized thresholds**.

Metrics	SUVmax	SUVpeak	SUVmean	SAM	MV	TLG	SULTAN
Threshold (%)	−36	−26	−29	−54	−58	−27	
Sensitivity (%)	75	72	67	39	8	53	83
Specificity (%)	98	98	95	98	100	100	88
PPV (%)	96	96	92	93	100	100	86
NPV (%)	82	81	77	66	57	72	86
Accuracy (%)	87	86	82	71	58	78	86%
Youden correlation coefficient	0.73	0.70	0.62	0.37	0.08	0.53	0.72
Significance (χ^2^* p* < 0.05)	S	S	S	NS	NS	S	S

**Figure 5:**
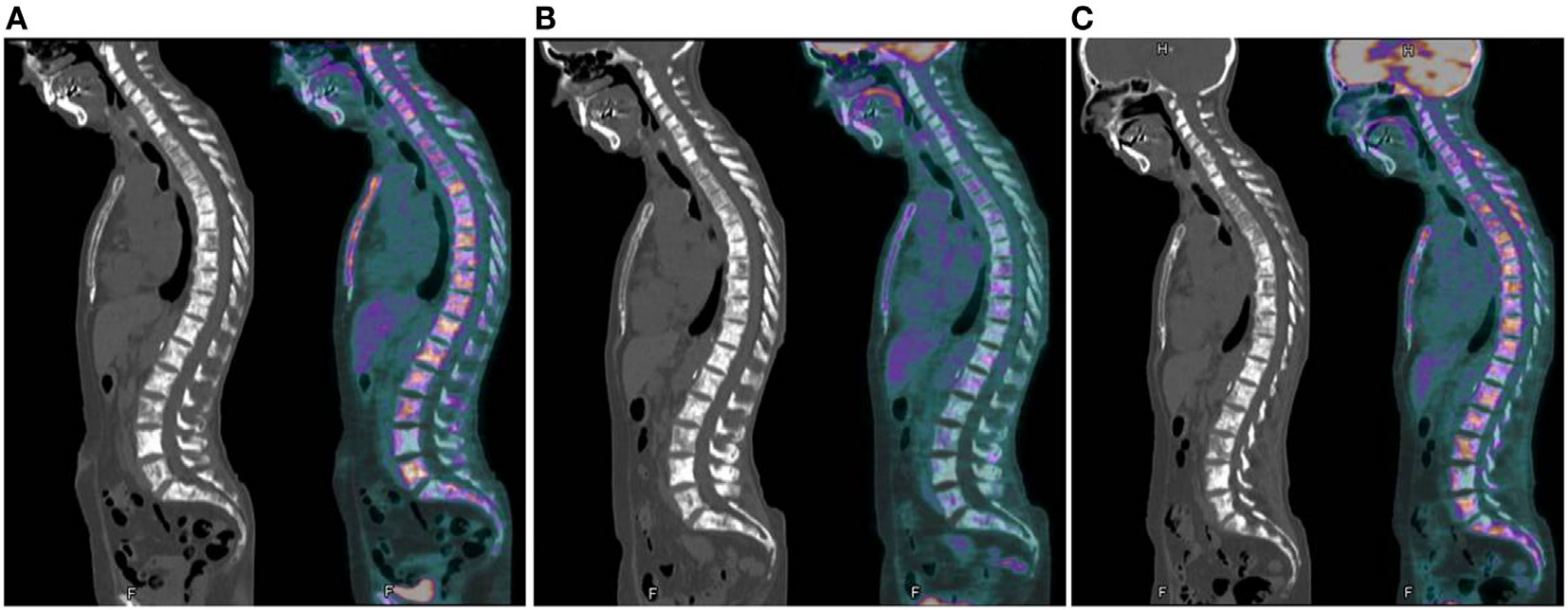
**Example of metabolic assessment in a patient with metastatic bone evolution**. **(A)** First examination: initial evaluation with multiple bone lesions (SUVmax = 11.8; SUVpeak = 7.1); **(B)** second examination: partial metabolic response on bone (SUVmax = 3.4 or 71% decrease; SUVpeak = 1.5 or 78% decrease); and **(C)** third examination: disease progression with new lesions and recurrence of some initial hypermetabolic lesions (SUVmax = 6.4 or 46% increase; SUVpeak = 4.3 or 65% increase). Persistence of sclerosis on all CT images does not allow to evaluate the response.

Figure 5 highlights the benefit of using quantitative PET-derived metrics for a metastatic bone patient. CT images failed to correctly classify the therapeutic response, with the persistence of an osteo-condensation even though there was a primary tumor response, thus highlighting the fact that bone lesions cannot be evaluated using RECIST 1.1.

### Per-Lesion and Per-Patient Analysis Using SULTAN

For the per-lesion analysis, results obtained with SULTAN (longitudinal monitoring in positron factor analysis) were compared with those obtained using SUVmax, SUVpeak, and SUVmean. No significant difference was found between the assessment of therapeutic response by the gold standard and SULTAN (*p* < 0.05).

**Figure 6:**
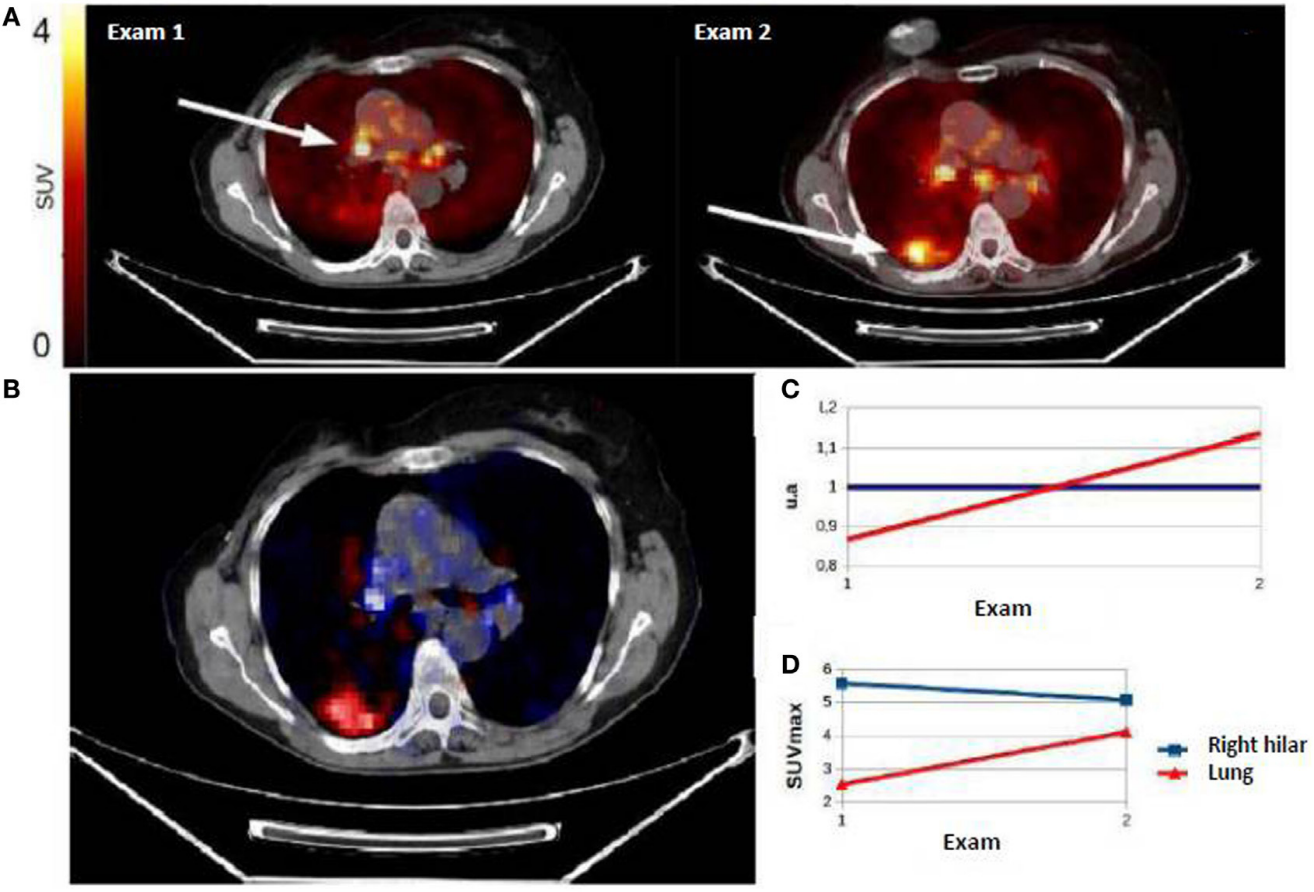
**(A)** Example of non-responder patient classified by SULTAN. First PET showed right hilar hypermetabolism, and second PET performed in therapeutic monitoring (exam 2) showed a progression with persistence of right hilar hypermetabolism and the appearance of a hypermetabolic right lung uptake. The evolution was classified as non-responder. Factorial image obtained by SULTAN was superimposed on the CT-scan 1 **(B)**. Associated curves **(C)** represented the growing trend (red) or stable (blue) voxels. The developments described by factor analysis were similar to those of SUVmax **(D)** with a stability of hilar fixation and the appearance of a right pulmonary uptake.

**Figure 7:**
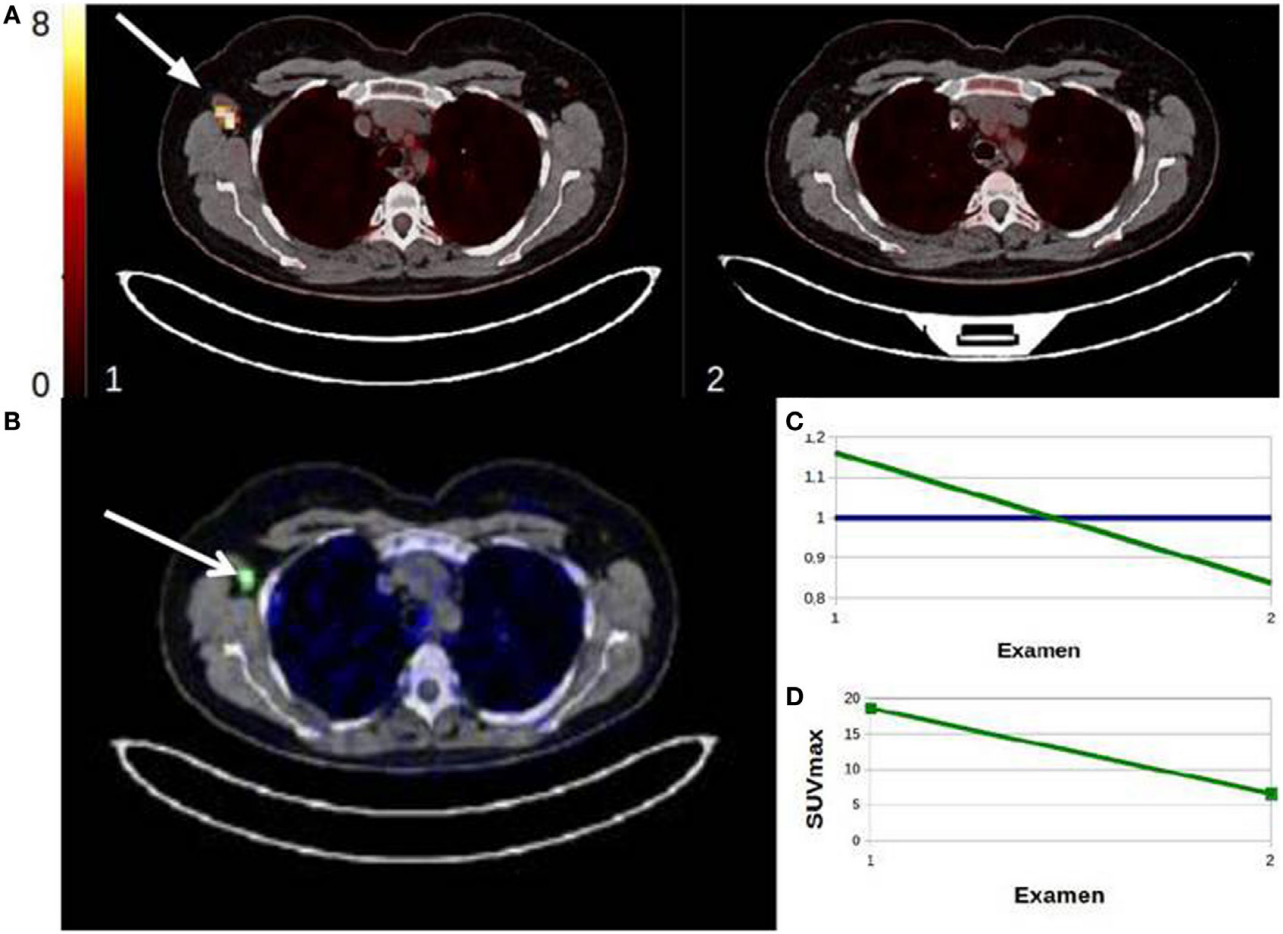
**Example of responder patient classified by SULTAN**. First PET showed right axillary lymph nodes hypermetabolism and the second PET, performed during therapeutic monitoring (review 2), showed a disappearance of the right axillary hypermetabolism. Factorial image obtained by SULTAN was superimposed on the CT-scan 1 **(B)**. Associated curves **(C)** represented the downward trend (green) or stable (blue) voxels. The developments described by factor analysis were similar to those of SUVmax **(D)** with a loss of the right axillary uptake.

For the per-patient PET analysis, SULTAN was compared with SUVmax, SUVpeak, and SUVmean, which appeared to be the only metrics significantly correlated to the gold standard. SULTAN presented no significant difference with SUVmax, SUVpeak, and SUVmean results using the PERCIST threshold (sensitivity: 83 vs. 75, 72, and 67%; NPV: 86 vs. 82, 81, and 77%, respectively). However, specificity and PPV were found to be lower than quantitative metrics (specificity: 88 vs. 98, 98, and 95%; PPV: 86 vs. 96, 96, and 92%) (Table 6). Figures 6 and 7 show an example of a responder and a non-responder patient using SULTAN.

**Table 6:** **Comparison of best metabolic metrics according to PERCIST and optimized thresholds and SULTAN for per-patient analysis**.

Metrics	SULTAN	SUVmax		SUVpeak		SUVmean	
		−30% PERCIST	−36%	−30% PERCIST	−26%	−30% PERCIST	−29%
Sensitivity (%)	83	75	75	67	72	67	67
Specificity (%)	88	95	98	98	98	95	95
PPV (%)	86	93	96	96	96	92	92
NPV (%)	86	82	82	78	81	77	77
Accuracy (%)	86	86	87	84	86	82	82
Youden correlation coefficient	0.72	0.70	0.73	0.64	0.70	0.62	0.62
Significance (χ^2^ *p* < 0.05)	S	S	S	S	S	S	S

## Discussion

Considering the limitations of morphological criteria and the subjectivity of visual analysis of metabolic imaging in the field of therapeutic evaluation, the use of quantitative PET-based metrics has gained interest in recent years (3, 7, 11–17, 26, 27). Depending on the disease studied, various metrics and thresholds have been established. In breast cancer, the majority of studies evaluating therapeutic response by metabolic metrics have been made in a neo-adjuvant setting, with histological confirmation, the true gold standard. In the adjuvant setting, the overall therapeutic response is usually assessed using morphological and metabolic imaging, and biological and clinical exams (7).

The choice of a preferred biomarker differs between neo-adjuvant and adjuvant settings. In the neo-adjuvant setting, with a curative intent, the NPV is the preferred relevant statistical information in early detection of non-responders before a change of therapy. In the adjuvant setting for metastatic patients, false-negative PET may lead to a treatment change. This was designed to counteract a false-positive that may lead to a reduced survival. In this situation, choosing the best couple sensitivity–specificity may be considered as an acceptable compromise.

It has been reported that a decrease of SUVmax or SUVmean after one or two cycles of chemotherapy was significantly correlated with a successful therapeutic response in the neo-adjuvant setting (7, 28–32). The optimal thresholds reported in these studies for discriminating responder and non-responder in per-patient analysis varied from 26 to 58%. These differences can be partly explained by the lack of consensus for the definition of responder and non-responder status (decrease in tumor mass >50% by histology or residual microscopic lesions), the population heterogeneity between studies (presence of hormone receptors, HER2 amplification, etc.), the time of PET completion (one, two, or three cycles of chemotherapy), and the criteria used to determine the best threshold.

However, only a few studies have used PET scans for evaluating the treatment response in the context of adjuvant therapy. Couturier et al. (15) showed that a decrease of SUVmax or SUVmean was predictive of therapeutic response after three cycles of chemotherapy using the same gold standard considered in our study. They speculated that response assessment using metabolic metrics appeared to be superior to visual analysis. The SUV decrease ranged from 52 to 56% for responders and 16 to 26% for non-responders. Dose Schwarz et al. (17) found that a SUVmax reduction of 72 ± 21% after one cycle and 54 ± 16% after two cycles of chemotherapy was predictive of response to treatment. Furthermore, Specht et al. (16) and Tateishi et al. (18) concluded that a decrease of SUVmean, and to a lesser extent of TLG for bone metastases, was predictive of the duration of response to treatment. In the study of Tateishi (18), a SUVmean decrease ≥8.5% was a factor significantly related to the duration of response, while the TLG did not. Huyge et al. (33) highlighted the significant heterogeneity of the metabolic response for the same patient when considering the types of metastases (bone or visceral). Using the change of SUVmax, according to the EORTC criteria, they highlighted a poorer therapeutic response for bone lesions. Finally, Quon and Gambhir (34) has warned that the “paradoxical metabolic flare,” which corresponds to an increase of SUV in the first 10 days after commencement of hormone therapy, may be misconstrued as a sign of an early metabolic reaction.

In our study, SUVmax, SUVpeak, and SUVmean were the most efficient metrics in the per-lesion and per-patient analysis. These observations are consistent with previously published results, which suggest the use of SUVmax (EORTC) or SULpeak (PERCIST). The SUVmax measurement is susceptible to be affected by noise due to its single-voxel determination (35). The use of SUVpeak may overcome this limitation and has been recommended as a more robust alternative due to its fixed volume of 1 cm^3^, therefore being less susceptible to noise than SUVmax. However, several definitions of SUVpeak are found in the literature differing in shape, size, and location of ROIpeak (36). As outlined in Section “Introduction,” many requirements imposed by the PERCIST criteria may be considered as too restrictive and difficult to apply in routine clinical situations. This is why we evaluated a “PERCIST-like” method with a SUV normalization against the mass of the patient (SUVpeak) rather than the lean body mass normalization (SULpeak) as recommended by PERCIST. The small size of the majority of measured lesions in our study, less than 2 cm, leads to a calculation of SUVpeak heavily weighted on SUVmax, thus explaining the high similarity of the results of the two indices.

The SUVmean index gave results similar to SUVmax and SUVpeak for the per-patient analysis, also explained by the small size of the measured lesions.

The SAM index was less efficient in our study and did not demonstrate benefit in our population. This index corresponds to the total excess SUV above the tumor background, reducing the impact of partial volume effect and lesion segmentation errors. Yet, Mertens et al. (22) reported good results with no significant difference with SUVmax in patients with colorectal cancer with progression to liver metastasis. The optimal threshold for differentiating responders and non-responders was set at 94.5 vs. 25.3% for the SUVmax, which is different from our results (54 vs. 36%).

Additionally, we showed that MV and TLG failed to correctly classify patients. In this respect, MV performance was variable: the approach to this calculation differs among centers with the use of gradients, thresholds, or adaptive method. In the neo-adjuvant therapy evaluation of breast cancer by ^18^F-FDG, Hatt et al. (37) found that TLG or MV, determined by a fuzzy locally adapted Bayesian algorithm, were better predictors than SUVmax, but the lesions they considered were larger than in our study. In our study, an adaptive method based on that described by Schaefer (23) was used, but it failed to correctly delineate the lesion when the signal-to-noise ratio was poor, explaining the poor ­performance of volume-based metrics.

Parametric imaging was found to be relevant in assessing the therapeutic response in breast cancer, with similar performance to SUVmax or SUVpeak. SULTAN has already been successfully assessed in patients with colorectal cancer and non-small lung cell carcinoma (20). SULTAN appears to be a valuable visual tool in routine clinical practice because of the otherwise tedious nature of measuring numerous lesions. Furthermore, using a single series of images, SULTAN provides a summary of all tumor evolutions from various scans without arbitrary threshold adjustment.

## Conclusion

Even if our study has limitations (heterogeneous population with patients in either first-line or advanced treatment, with varied histological and phenotypic characteristics and different treatments), the results underline the importance of the metrics choice for PET evaluation. SUVmax, SUVpeak, and to a lesser extent SUVmean appeared to be the most relevant metrics. In addition, parametric analysis using the SULTAN approach is a reliable tool to guide visual interpretation. The poor performances of volumetric metrics underline the need for developing and validating a robust delineation method that could be applied in the context of small lesion with a poor signal-to-noise ratio. In the future, a comparison of metrics could be conducted in a prospective study performed in a homogeneous population.

## Author Contributions

DG: data measure and paper writing; HN: statistical analysis and parametric imaging; BH: statistical analysis; CR: patient recruitment; and TC and FK-B: study conception and paper correction.

## Conflict of Interest Statement

The authors declare that the research was conducted in the absence of any commercial or financial relationships that could be construed as a potential conflict of interest.

## Abbreviations

AUC, area under curve; EORTC, European Organization for Research and Treatment of Cancer; FADS, factor analysis of dynamic sequences; FDG, fluorodeoxyglucose; FN, false negative; FP, false positive; MRI, magnetic resonance imaging; MV, metabolic volume; NPV, negative predictive value; PET, positron emission tomography; PPV, positive predictive value; ROC, receiver operating characteristic; ROI, region of interest; SAM, standardized added metabolic activity; SULTAN, longitudinal monitoring in positron factor analysis; SUL, SUV lean body mass; SUV, standard uptake value; TLG, total lesion glycolysis; TN, true negative; TP, true positive.

## References

[1] Luporsi E Le cancer du sein métastatique. Définitions actuelles, épidémiologie, présentations cliniques. Springer (2007). Available from: http://documents.irevues.inist.fr/bitstream/handle/2042/15908/1/SFSPM_2007_17.pdf

[2] Eisenhauer EA Therasse P Bogaerts J Schwartz LH Sargent D Ford R New response evaluation criteria in solid tumours: revised RECIST guideline (version 1.1). Eur J Cancer (2009) 45:228–47. 10.1016/j.ejca.2008.10.026 19097774

[3] Wahl RL Jacene H Kasamon Y Lodge MA. From RECIST to PERCIST: evolving considerations for PET response criteria in solid tumors. J Nucl Med (2009) 50(Suppl 1):122S–50S. 10.2967/jnumed.108.057307 19403881 PMC2755245

[4] Carlier T Bailly C. State-of-the-art and recent advances in quantification for therapeutic follow-up in oncology using PET. Front Med (2015) 2:18. 10.3389/fmed.2015.00018 26090365 PMC4370108

[5] Cachin F Kelly A Maublant J Evaluation of the therapeutic response: role of isotopic imaging. Bull Cancer (2006) 93:1191–9. 10.1684/bdc.2006.0145 17182375

[6] Young H Baum R Cremerius U Herholz K Hoekstra O Lammertsma AA Measurement of clinical and subclinical tumour response using [18F]-fluorodeoxyglucose and positron emission tomography: review and 1999 EORTC recommendations. European Organization for Research and Treatment of Cancer (EORTC) PET Study Group. Eur J Cancer (1999) 35:1773–82. 10.1016/S0959-8049(99)00229-4 10673991

[7] Groheux D Espié M Giacchetti S Hindié E. Performance of FDG PET/CT in the clinical management of breast cancer. Radiology (2013) 266:388–405. 10.1148/radiol.12110853 23220901

[8] Groheux D Moretti J-L Baillet G Espie M Giacchetti S Hindie E Effect of (18)F-FDG PET/CT imaging in patients with clinical stage II and III breast cancer. Int J Radiat Oncol Biol Phys (2008) 71:695–704. 10.1016/j.ijrobp.2008.02.056 18436392

[9] Pan L Han Y Sun X Liu J Gang H. FDG-PET and other imaging modalities for the evaluation of breast cancer recurrence and metastases: a meta-analysis. J Cancer Res Clin Oncol (2010) 136:1007–22. 10.1007/s00432-009-0746-6 20091186 PMC2874488

[10] Pennant M Takwoingi Y Pennant L Davenport C Fry-Smith A Eisinga A A systematic review of positron emission tomography (PET) and positron emission tomography/computed tomography (PET/CT) for the diagnosis of breast cancer recurrence. Health Technol Assess Winch Engl (2010) 14:1103 10.3310/hta14500 21044553

[11] Wahl RL Zasadny K Helvie M Hutchins GD Weber B Cody R. Metabolic monitoring of breast cancer chemohormonotherapy using positron emission tomography: initial evaluation. J Clin Oncol (1993) 11:2101–11. 8229124 10.1200/JCO.1993.11.11.2101

[12] Avril S Muzic RF Jr Plecha D Traughber BJ Vinayak S Avril N. 18F-FDG PET/CT for monitoring of treatment response in breast cancer. J Nucl Med (2016) 57(Suppl 1):34S–9S. 10.2967/jnumed.115.157875 26834099 PMC5228521

[13] Groheux D Mankoff D Espié M Hindié E F-FDG PET/CT in the early prediction of pathological response in aggressive subtypes of breast cancer: review of the literature and recommendations for use in clinical trials. Eur J Nucl Med Mol Imaging (2016) 43:983–93. 10.1007/s00259-015-3295-z 26758726

[14] Lin NU Guo H Yap JT Mayer IA Falkson CI Hobday TJ Phase II study of lapatinib in combination with trastuzumab in patients with human epidermal growth factor receptor 2-positive metastatic breast cancer: clinical outcomes and predictive value of early [18F]fluorodeoxyglucose positron emission tomography imaging (TBCRC 003). J Clin Oncol (2015) 33:2623–31. 10.1200/JCO.2014.60.0353 26169615 PMC4534525

[15] Couturier O Jerusalem G N’Guyen J-M Hustinx R. Sequential positron emission tomography using [18F]fluorodeoxyglucose for monitoring response to chemotherapy in metastatic breast cancer. Clin Cancer Res (2006) 12:6437–43. 10.1158/1078-0432.CCR-06-0383 17085657

[16] Specht JM Tam SL Kurland BF Gralow JR Livingston RB Linden HM Serial 2-[18F] fluoro-2-deoxy-D-glucose positron emission tomography (FDG-PET) to monitor treatment of bone-dominant metastatic breast cancer predicts time to progression (TTP). Breast Cancer Res Treat (2007) 105:87–94. 10.1007/s10549-006-9435-1 17268819

[17] Dose Schwarz J Bader M Jenicke L Hemminger G Jänicke F Avril N. Early prediction of response to chemotherapy in metastatic breast cancer using sequential 18F-FDG PET. J Nucl Med (2005) 46:1144–50. 16000283

[18] Tateishi U Gamez C Dawood S Yeung HWD Cristofanilli M Macapinlac HA. Bone metastases in patients with metastatic breast cancer: morphologic and metabolic monitoring of response to systemic therapy with integrated PET/CT. Radiology (2008) 247:189–96. 10.1148/radiol.2471070567 18372468

[19] Necib H Garcia C Wagner A Vanderlinden B Emonts P Hendlisz A Detection and characterization of tumor changes in 18F-FDG PET patient monitoring using parametric imaging. J Nucl Med (2011) 52:354–61. 10.2967/jnumed.110.080150 21345787

[20] Necib H Characterization of the Tumor Changes During the Course of Therapy Using PET/CT Scans. Paris: University of Paris Sud 11 (2009).

[21] Necib H Dusart M Tylski P Vanderlinden B Buvat I Detection of the tumor changes between two FDG PET scans using parametric imaging. J Nucl Med (2008) 49(Suppl 1):121.

[22] Mertens J De Bruyne S Van Damme N Smeets P Ceelen W Troisi R Standardized added metabolic activity (SAM) IN ^18^F-FDG PET assessment of treatment response in colorectal liver metastases. Eur J Nucl Med Mol Imaging (2013) 40:1214–22. 10.1007/s00259-013-2421-z 23636802

[23] Schaefer A Kremp S Hellwig D Rübe C Kirsch C-M Nestle U. A contrast-oriented algorithm for FDG-PET-based delineation of tumour volumes for the radiotherapy of lung cancer: derivation from phantom measurements and validation in patient data. Eur J Nucl Med Mol Imaging (2008) 35:1989–99. 10.1007/s00259-008-0875-1 18661128

[24] Vauclin S Doyeux K Hapdey S Edet-Sanson A Vera P Gardin I. Development of a generic thresholding algorithm for the delineation of 18FDG-PET-positive tissue: application to the comparison of three thresholding models. Phys Med Biol (2009) 54:6901–16. 10.1088/0031-9155/54/22/010 19864698

[25] Frouin F Bazin JP Di Paola M Jolivet O Di Paola R. FAMIS: a software package for functional feature extraction from biomedical multidimensional images. Comput Med Imaging Graph (1992) 16(2):81–91. 10.1016/0895-6111(92)90121-O 1568204

[26] Rousseau C Devillers A Sagan C Ferrer L Bridji B Campion L Monitoring of early response to neoadjuvant chemotherapy in stage II and III breast cancer by [18F]fluorodeoxyglucose positron emission tomography. J Clin Oncol (2006) 24:5366–72. 10.1200/JCO.2006.05.7406 17088570

[27] Wang Y Zhang C Liu J Huang G. Is 18F-FDG PET accurate to predict neoadjuvant therapy response in breast cancer? A meta-analysis. Breast Cancer Res Treat (2012) 131:357–69. 10.1007/s10549-011-1780-z 21960111

[28] Schwarz-Dose J Untch M Tiling R Sassen S Mahner S Kahlert S Monitoring primary systemic therapy of large and locally advanced breast cancer by using sequential positron emission tomography imaging with [18F]fluorodeoxyglucose. J Clin Oncol (2009) 27:535–41. 10.1200/JCO.2008.17.2650 19075273

[29] Schelling M Avril N Nährig J Kuhn W Römer W Sattler D Positron emission tomography using [(18)F]fluorodeoxyglucose for monitoring primary chemotherapy in breast cancer. J Clin Oncol (2000) 18:1689–95. 10764429 10.1200/JCO.2000.18.8.1689

[30] McDermott GM Welch A Staff RT Gilbert FJ Schweiger L Semple SIK Monitoring primary breast cancer throughout chemotherapy using FDG-PET. Breast Cancer Res Treat (2007) 102:75–84. 10.1007/s10549-006-9316-7 16897427

[31] Duch J Fuster D Muñoz M Fernández PL Paredes P Fontanillas M PET/CT with [18F] fluorodeoxyglucose in the assessment of metabolic response to neoadjuvant chemotherapy in locally advanced breast cancer. Q J Nucl Med Mol Imaging (2012) 56(3):291–8. 22695339

[32] Berriolo-Riedinger A Touzery C Riedinger J-M Toubeau M Coudert B Arnould L [18F]FDG-PET predicts complete pathological response of breast cancer to neoadjuvant chemotherapy. Eur J Nucl Med Mol Imaging (2007) 34:1915–24. 10.1007/s00259-007-0459-5 17579854

[33] Huyge V Garcia C Alexiou J Ameye L Vanderlinden B Lemort M Heterogeneity of metabolic response to systemic therapy in metastatic breast cancer patients. Clin Oncol (R Coll Radiol) (2010) 22:818–27. 10.1016/j.clon.2010.05.021 20554438

[34] Quon A Gambhir SS FDG-PET and beyond: molecular breast cancer imaging. J Clin Oncol (2005) 23:1664–73. 10.1200/JCO.2005.11.024 15755974

[35] Boellaard R Krak NC Hoekstra OS Lammertsma AA. Effects of noise, image resolution, and ROI definition on the accuracy of standard uptake values: a simulation study. J Nucl Med (2004) 45:1519–27. 15347719

[36] Vanderhoek M Perlman SB Jeraj R. Impact of the definition of peak standardized uptake value on quantification of treatment response. J Nucl Med (2012) 53:411. 10.2967/jnumed.111.093443 22213818 PMC3308343

[37] Hatt M Groheux D Martineau A Espié M Hindié E Giacchetti S Comparison between 18F-FDG PET image-derived indices for early ­prediction of response to neoadjuvant chemotherapy in breast cancer. J Nucl Med (2013) 54:341–9. 10.2967/jnumed.112.108837 23327900

